# Tomato SPAK and OFPs act as promising targets for fine-tuning fruit elongation by affecting cell division patterns at the proximal end

**DOI:** 10.1093/hr/uhag123

**Published:** 2026-04-07

**Authors:** Qiang Li, Bingbing Cai, Fubin Zhang, Yike Liu, Yunuo Chang, Xiao Du, Xupeng Jia, Esther van der Knaap, Qingyun Li

**Affiliations:** College of Horticulture, Key Laboratory of Vegetable Germplasm Innovation and Utilization of Hebei, Baoding, Hebei Province 071000, China; College of Horticulture, Key Laboratory of Vegetable Germplasm Innovation and Utilization of Hebei, Baoding, Hebei Province 071000, China; College of Horticulture, Key Laboratory of Vegetable Germplasm Innovation and Utilization of Hebei, Baoding, Hebei Province 071000, China; College of Horticulture, Key Laboratory of Vegetable Germplasm Innovation and Utilization of Hebei, Baoding, Hebei Province 071000, China; College of Horticulture, Key Laboratory of Vegetable Germplasm Innovation and Utilization of Hebei, Baoding, Hebei Province 071000, China; College of Horticulture, Key Laboratory of Vegetable Germplasm Innovation and Utilization of Hebei, Baoding, Hebei Province 071000, China; College of Horticulture, Key Laboratory of Vegetable Germplasm Innovation and Utilization of Hebei, Baoding, Hebei Province 071000, China; Center for Applied Genetic Technologies, University of Georgia, Athens, GA 30602, USA; Department of Horticulture, University of Georgia, Athens, GA 30602, USA; College of Horticulture, Key Laboratory of Vegetable Germplasm Innovation and Utilization of Hebei, Baoding, Hebei Province 071000, China

Dear Editor,

The shape of fruit serves as a pivotal determinant of quality, yield, harvesting method and consumer preference, and is integral to the cultivation of fruit-bearing crops. The fruit proximal end plays a crucial role in determining fruit shape. In peppers, this area is essential in classifying market varieties, such as long linear and horn peppers. For cucumbers, the proximal end forms the fruit neck, negatively influencing both fruit length and commercial value [1]. Therefore, elucidating the molecular foundations governing the shape of the proximal region is of great importance for advancing crop improvement.

OVATE Family Proteins (OFPs) and TONNEAU1 Recruiting Motif proteins (TRMs) are well known as key regulators of fruit elongation by influencing cell division patterns at the proximal end [2, 3]. Identifying and characterizing additional genes regulating fruit shape through the proximal end is crucial for understanding regulatory networks and facilitating fruit shape improvement in future. Never in mitosis A-related kinases (NEKs) is a family of serine/threonine kinases that have been reported to be associated with cell-cycle regulation. Tomato *SELF-PRUNING* (SP)-*associated kinase* (*SPAK*, *Solyc01g097500*) was a member of NEK family, and down-regulation of *SPAK* leads to elongated fruits [4]. However, the detailed functions of SPAK in fruit shape control are still largely unknown.

The colocalization of the SPAK-RFP with the microtubule marker protein GFP-MAP4 indicated the microtubule localization of SPAK (Fig. 1A). Furthermore, the application of oryzalin led to the disruption of microtubules labeled with both SPAK-RFP and GFP-MAP4, further supporting SPAK’s association with microtubules (Fig. 1A). Two knock-out mutants of SPAK, *spak-1* and *spak-2*, were created. The fruits of the *spak-1* and *spak-2* single mutants were notably longer and much slender than those of the wild type (WT) (Fig. 1B–F), indicating the negative roles of *SPAK* in regulating fruit elongation. We further investigated the proximal end angle (PA) and distal end angle (DA), which are important attributes of fruit shape. The *spak-1* and *spak-2* displayed a notably reduced PA in comparison with the WT, although the DA showed no significant variation between WT and the *SPAK* knockout lines (Fig. 1G–H). Given that the fruit shape of *spak-1* and *spak-2* mutants was comparable, *spak-2* was chosen for further examinations.

**Figure 1:**
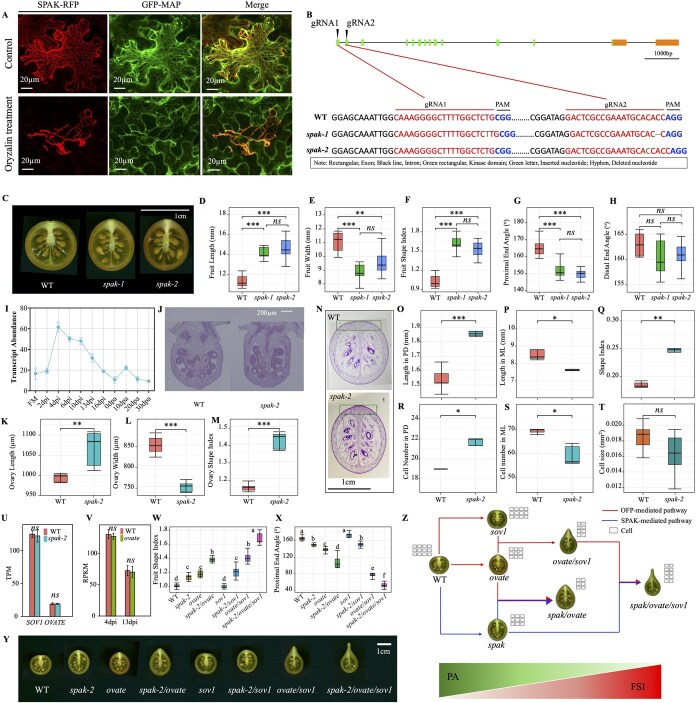
Tomato SPAK and OFPs can fine-tune fruit elongation at the proximal end. (A) Subcellular localization of SPAK; (B) gene structure and CRISPR-generated mutations of *SPAK*; (C) Representative fruits at breaker stage; (D–H) comparisons of fruit shape attributes; (I) Transcript abundance of *SPAK* during flower and fruit development; (J) paraffin sections of representative ovaries in WT and *spak-2*; (K–M) comparisons of ovary length, width and shape index between WT and *spak-2*; (N) paraffin sections of representative fruits at breaker stage in WT and *spak-2*; (O–Q) comparisons of length in PD, length in ML and shape index of proximal region between WT and *spak-2*; (R–T) comparisons of cell numbers in PD and ML as well as cell sizes at the proximal end between WT and *spak-2*; (U) expression analyses of *OVATE* and *SOV1* in WT and *spak-2*; (V) expression analyses of *SPAK* in WT and *ovate*; (W, X) comparisons of FSI and PA among various NILs; The lowercase letters indicate the difference of *P* < 0.05 by Duncan’s test; (Y) representative fruits at breaker stage in various NILs; (Z) schematic diagram of SPAK and OFPs in fine-tuning fruit elongation by affecting cell division patterns at the proximal end. Asterisks denote significant differences (^*^P < 0.05; ^**^P < 0.01; ^***^P < 0.001) as determined by Student’s *t*-tests. The fruit shape attributes were measured using Tomato Analyzer 4.0. ns, nonsignificant difference; FM, floral meristem; dpi, days postflower initiation; dpa, days post anthesis; PD, proximal-distal direction; ML, medio-lateral direction; PA, proximal end angle; FSI, fruit shape index; PAM, Protospacer Adjacent Motif.

The analysis of gene expression during various flower and fruit developmental stages revealed that *SPAK* is prominently expressed prior to anthesis (Fig. 1I). Importantly, the *spak-2* exhibited notable alterations in ovary shape at anthesis, with increases in ovary length and shape index, alongside a reduction in ovary width (Fig. 1J–M), suggesting that SPAK influences fruit shape prior to anthesis.

We further explore the morphological changes at the proximal end of fruits at breaker stage. As expected, the proximal end of the fruits in *spak-2* mutant was significantly narrower and longer than that of WT, leading to significantly increased shape index compared to that of WT (Fig. 1N–Q). Compared to WT, significantly more cells in the proximal-distal direction and less cells in the mediolateral direction were found in the proximal region of fruits in *spak-2* (Fig. 1R–S). However, the cell sizes in the proximal region of fruits were similar between *spak-2* and WT (Fig. 1T). These results suggested that the elongated fruit proximal end was due to the changes of the cell division patterns, not cell size, driven by SPAK. This altered cell division pattern in *spak-2* may arise from either an increased cell division rate in the longitudinal direction, or a higher frequency of transverse cell divisions.

Considering the similar functions in regulating fruit shape between the SPAK and OVATE [5], we investigated the interaction between them. Nevertheless, no interaction was detected between SPAK and OVATE or SOV1 using yeast-two-hybrid (data not shown). Notably, the expression of *OVATE* and *SOV1* exhibited no significant difference between WT and *spak-2*, while *SPAK* expression also showed no significant variation between WT and *ovate* (Fig. 1U and V). We subsequently explored the genetic interactions between SPAK and the two OFPs. Compared to the effects of *spak-2* in WT background, introduction of *spak-2* into the *ovate*, *sov1*, and *ovate*/*sov1* backgrounds lead to significantly increased fruit shape index and decreased PA (Fig. 1W–Y). Notably, the pyramiding line *spak-2/ovate/sov1* exhibited a much longer and narrower proximal region compared with other single and double NILs in LA1589 background (Fig. 1W–Y). These results suggest that SPAK works additively with OVATE and SOV1 in negative regulation of fruit elongation in tomato.

In conclusion, we demonstrated that SPAK encodes a microtubule-associated protein and functions as a negative regulator influencing fruit elongation prior to anthesis in tomato. The fruit elongation caused by *SPAK* null alleles is attributed to increased length of the fruit proximal end by influencing cell division patterns without affecting cell size. SPAK governs fruit elongation additively with OVATE and SOV1. These findings provide novel insights into the role of SPAK in fruit shape regulation and propose its potential application for fine-tuning fruit elongation in crops (Fig. 1Y and Z).

## Data Availability

Transcriptome data from ovaries at 0DPA of WT and *spak-2* have been archived in the China National Center for Bioinformation (CNCB) under project accession PRJCA059071. The data supporting the findings of this study are available from the corresponding authors upon request.
